# In vivo compartmental kinetics of *Plasmodium falciparum* histidine-rich protein II in the blood of humans and in BALB/c mice infected with a transgenic *Plasmodium berghei* parasite expressing histidine-rich protein II

**DOI:** 10.1186/s12936-019-2712-3

**Published:** 2019-03-13

**Authors:** Kristin E. Poti, Amanda E. Balaban, Priya Pal, Tamaki Kobayashi, Daniel E. Goldberg, Photini Sinnis, David J. Sullivan

**Affiliations:** 1 Department of Molecular Microbiology and Immunology, Johns Hopkins Bloomberg School of Public Health, Baltimore, MD USA; 2 Johns Hopkins Malaria Research Institute, Johns Hopkins Bloomberg School of Public Health, Baltimore, MD USA; 3 Division of Infectious Diseases, Department of Medicine, Washington University School of Medicine, St. Louis, MO USA; 4 Department of Molecular Microbiology, Washington University School of Medicine, St. Louis, MO USA; 5 Department of Epidemiology, Johns Hopkins Bloomberg School of Public Health, Baltimore, MD USA

**Keywords:** Malaria, Histidine-rich protein II, HRP2, Persistence, Once-infected RBCs, Erythrocyte pitting, *Plasmodium berghei*, Rapid diagnostic test

## Abstract

**Background:**

The *Plasmodium falciparum* histidine-rich protein II (PfHRP2) is a common biomarker used in malaria rapid diagnostic tests (RDTs), but can persist in the blood for up to 40 days following curative treatment. The persistence of PfHRP2 presents a false positive limitation to diagnostic interpretation. However, the in vivo dynamics and compartmentalization underlying PfHRP2 persistence have not been fully characterized in the plasma and erythrocyte (RBC) fraction of the whole blood.

**Methods:**

The kinetics and persistence of PfHRP2 in the plasma and RBC fractions of the whole blood were investigated post-treatment in human clinical samples and samples isolated from BALB/c mice infected with a novel transgenic *Plasmodium berghei* parasite engineered to express PfHRP2 (PbPfHRP2).

**Results:**

PfHRP2 levels in human RBCs were consistently 20–40 times greater than plasma levels, even post-parasite clearance. PfHRP2 positive, DNA negative, once-infected RBCs were identified in patients that comprised 0.1–1% of total RBCs for 6 and 12 days post-treatment, even post-atovaquone–proguanil regimens. Transgenic PbPfHRP2 parasites in BALB/c mice produced and exported tgPfHRP2 to the RBC cytosol similar to *P. falciparum*. As in humans, tgPfHRP2 levels were found to be approximately 20-fold higher within the RBC fraction than the plasma post-treatment. RBC localized tgPfHRP2 persisted longer than tgPfHRP2 in the plasma after curative treatment. tgPfHRP2 positive, but DNA negative once-infected RBCs were also detected in mouse peripheral blood for 7–9 days after curative treatment.

**Conclusions:**

The data suggest that persistence of PfHRP2 is due to slower clearance of protein from the RBC fraction of the whole blood. This appears to be a result of the presence PfHRP2 in previously infected, pitted cells, as opposed to PfHRP2 binding naïve RBCs in circulation post-treatment. The results thus confirm that the extended duration of RDT positivity after parasite clearance is likely due to pitted, once-infected RBCs that remain positive for PfHRP2.

**Electronic supplementary material:**

The online version of this article (10.1186/s12936-019-2712-3) contains supplementary material, which is available to authorized users.

## Background

As of 2017, there were over 200 million malaria cases and 435,000 deaths [1]. Greater than 90% of these malaria infections were caused by the *Plasmodium falciparum* parasite predominately in Africa, which causes the most severe disease in humans [1]. In Africa, most malaria rapid diagnostic tests (RDT) detect the abundant *P. falciparum* protein, histidine-rich protein II (PfHRP2) [2–4]. Other RDTs detect aldolase or lactate dehydrogenase either alone or in combination. At parasite densities greater than 500–1000 parasites/µL of whole blood, the RDT has demonstrated high sensitivity and specificity, similar to the microscopy standard [3–6]. Additionally, the low cost, minimal resources required, rapid results, and ease of use, has led the World Health Organization to recommend the RDT for diagnosing malaria prior to treatment in the field [7].

Despite the use of PfHRP2 as the antigen in the RDT, PfHRP2 compartmental kinetics in the two blood fractions during infection and during parasite clearance post-treatment have not been extensively characterized. In particular, the dynamics of PfHRP2 in the RBC fraction of the whole blood has not been specifically measured. To date, the half-life of PfHRP2 has been estimated to be 3.67 days in plasma and 3–5 days in the whole blood following treatment [8, 9]. Ndour et al. also demonstrated that overall PfHRP2 levels in whole blood treatment decreased at a slower rate compared to PfHRP2 plasma levels when compared only on day 0 and 3 post-treatment [10]. However, no estimations were specifically determined for PfHRP2 clearance time from the RBC fraction of the whole blood after treatment for comparison, nor were these kinetic parameters in the two different blood fractions (plasma *vs*. RBCs) compared to the clearance time of parasite genomic DNA. Additionally, PfHRP2 demonstrates a unique biologic persistence in patients regardless of successful treatment with anti-malarials, indicating that PfHRP2 clears from the blood slower than parasites [10–13]. In fact, PfHRP2 can persist at low, detectable levels for several weeks after treatment [10–13], presenting a challenge for RDT interpretation as a result of false positive interpretations of test results, especially when utilizing these tests in malaria endemic regions or to measure treatment response and assess treatment failure [14].

*Plasmodium falciparum* is the only human *Plasmodium* species that produces PfHRP2 with expression throughout the asexual intraerythrocytic cycle and early stages of gametocytogenesis [15–21]. Each parasite produces approximately 2–10 femtograms of PfHRP2 per 48 h replication cycle [20, 21], but only 3% of PfHRP2 produced is retained by the parasite, localizing to the parasite cytoplasm or food vacuole [22, 23]. The remaining 97% is exported into the infected RBC (iRBC) cytoplasm, concentrating in packets near the iRBC membrane [16, 22]. PfHRP2 can also be secreted from intact iRBCs, but the majority (> 90%) is released when mature schizonts rupture the iRBC to release merozoites [16, 19]. Following release, PfHRP2 can be detected in various bodily fluids including whole blood, RBCs, plasma, serum, urine, cerebral spinal fluid, and saliva [16, 19, 24–27]. However, the physiological function of PfHRP2 and the significance of its localization are unknown. Experimental evidence indicates PfHRP2 can promote coagulation [28], interfere with immune cell activation [29], and may contribute to cerebral malaria pathology by disrupting barrier integrity in the brain [30].

To probe PfHRP2 kinetics in vivo, human whole blood samples were fractionated into their component parts of plasma and washed RBCs for PfHRP2 quantification and immunofluorescence analysis. A novel transgenic *Plasmodium berghei* mouse parasite was also engineered to express the PfHRP2 protein (PbPfHRP2) for an additional in vivo model for further correlative studies of PfHRP2 kinetics.

## Methods

### *Plasmodium falciparum* patient blood collection

De-identified, human whole blood samples isolated from patients with a *P. falciparum* infection were collected from the Johns Hopkins Hospital Microbiology Lab. DNA was extracted from 20 µL of whole blood using the QIAamp DNA Blood Mini Kit and eluted in 50 µL of water for qPCR amplification, as described previously [31], using novel oligonucleotide primers and a fluorescent probe specific for the *P. falciparum* Pfs25 gene (Additional file 1: Table S1). The remaining blood was centrifuged at 5000×*g* for 4.5 min to separate plasma and RBC fractions. The RBCs were washed four times with 1 mL of PBS.

### Recombinant and native PfHRP2 protein purification

The PfHRP2 gene, inserted into the pET 15b vector and cloned into *Escherichia coli* BL 21/DE3 cells, was expressed and purified using a previously described protocol [23]. Native PfHRP2 protein was purified using the same protocol from pelleted RBCs cultured with the *P. falciparum* FCQ79 parasite strain provided by PATH [23]. Concentrations of purified proteins were determined using amino acid analysis completed by the Protein Structure Core at the University of Nebraska.

### Construction of transgenic PbPfHRP2 parasite

The full-length *P. falciparum* 3D7 HRP2 (PF3D7_0831800) gene was inserted into the pL1694 vector and expressed under the *P. berghei* HSP70 promoter, with a 2A skip peptide between PfHRP2 and GFP. The cassette was flanked with integration sites for the 230p gene in *P. berghei*. Second passage *P. berghei* (strain ANKA) parasites were purified from 5 mice at 1% parasitaemia by collecting blood via cardiac puncture. Purified, mature schizonts (5 × 10^7^) were generated in vitro and transfected with 10 µg of pL1694 + HRPII-2A-GFP linearized with Bcl1 and Sap1, as previously described [32, 33]. Transfected parasites were injected into the tail vein of 6–8-week-old female Swiss Webster mice (Taconic). Utilizing the human dihydrofolate reductase (DHFR) selection cassette, progeny were selected with pyrimethamine at 7 mg/mL in the drinking water. Blood was collected via cardiac puncture and single clones of transfected parasites were generated by diluting parasites at 1 parasite/mouse across 20 mice. Isolated clones were screened by PCR for correct genetic manipulation (Additional file 2). PfHRP2 protein expression in vivo was verified by RDT, western blot, and immunofluorescence microscopy (Fig. 3).

### In vivo murine model for measuring transgenic PfHRP2 kinetics

Eleven 8-week-old female BALB/c mice (Jackson Laboratory) were infected with approximately 500,000 to 1,000,000 PbPfHRP2 iRBCs isolated from a donor mouse. Once mice reached > 5% parasitaemia at days 4–5 post-infection, they were administered, via intraperitoneal injection, 3 doses of 50 mg/kg of artesunate at 0, 8, and 24 h and a single dose of 36 mg/kg of pyronaridine at 24 h. Artesunate (Sigma-Aldrich) was dissolved in 5% NaHCO_3_ and pyronaridine tetraphosphate (Sigma-Aldrich) was dissolved in nuclease-free water. Parasite clearance time was monitored by blood films and qPCR performed as previously described [31] on DNA extracted from 20 µL of whole blood, using primers and probes specific for *P. berghei ANKA* 18S rRNA (Additional file 1: Table S1). Three copies of 18S rRNA/parasite was assumed to calculate parasites/µL because the primers and probes are capable of binding 3 copies of the *P. berghei* 18S rRNA gene on chromosomes 5, 7 and 12, as was determined by BLASTing the sequences of these primers and probes against the *P. berghei* ANKA genome using PlasmoDB v.41. In the *P. berghei* genome, there are four ribosomal units, classified into two types, A-type (includes units A and B) and S-type (includes units C and D) [34], and three of these four units have been shown to have greater than 90% sequence homology (A, C, and D) [34–36]. 140 µL of tail blood was drawn daily for protein quantification. Drawing this volume of blood daily did not impact protein kinetics or clearance, as blood drawn only prior to treatment (day 0) and on day 5 or 7 post-treatment yielded similar PfHRP2 kinetics.

The blood was centrifuged at 5000×*g* for 4.5 min to separate plasma and RBC fractions. The RBCs were washed four times with 1 mL of PBS. The same methods were used for the splenic and asplenic mouse experiments. Female BALB/c mice (n = 5) were splenectomized by a Johns Hopkins veterinarian at 7 weeks of age, 2 weeks prior to infection.

### Immunofluorescence microscopy

Slides were fixed and permeabilized in methanol at − 20 °C for 20 min and stored at − 80 °C. Prior to staining, slides were blocked for 1 h at room temperature with 1% bovine serum albumin (BSA)/PBS. Slides were washed with PBS and incubated for 1 h with mouse anti-PfHRP2 antibody 3A4 (1 mg/mL) conjugated to Alexa Fluor-594 diluted 1:200 in 1% BSA/PBS. After washing, slides were incubated with a rabbit anti-*Plasmodium* enolase antibody diluted 1:50 in 1% BSA/PBS. Slides were washed and incubated for 1 h with Alexa Fluor-488 labeled goat anti-rabbit IgG (Invitrogen), diluted 1:500 in 1% BSA/PBS. Slides were washed and briefly dried. ProLong Gold antifade reagent with DAPI (ThermoFisher) was added to the wells and sealed under a coverslip. Images were captured with the Zeiss AxioImager M2 microscope equipped with a Hamamatsu Orca R2 camera. Deconvolution and image analysis were done using the Velocity Imaging Software. Approximately 1500 RBCs were counted for each human patient (n = 6) to determine the percentage of PfHRP2 positive, DAPI negative, once-infected RBCs.

### PfHRP2 enzyme-linked immunosorbent assay (ELISA)

The Malaria Ag CELISA specific for PfHRP2 used to quantify PfHRP2 in murine and patient samples was performed according to the manufacturer’s instructions [37]. Absorbance was read at 450 nm using the BMG Labtech POLARstar OPTIMA plate reader. The limit of detection (LOD) was determined on each plate by averaging the absorbance of the negative controls for each blood fraction and adding 3 standard deviations to the mean. Any absorbance reading under the LOD was defined as PfHRP2 negative. The limit of quantitation (LOQ) was determined by the linear range of the standard curve on each plate. The LOQ was approximately 2 ng/mL of PfHRP2.

### PfHRP2 RBC binding assay

Plasma isolated from *P. falciparum*-infected patients (n = 5) at a concentration of 1000–3000 ng/mL of PfHRP2, and human serum spiked with a very high concentration of rPfHPR2 well above circulating plasma levels observed during severe infection, or a lower concentration of PfHRP2, were added to uninfected, human O^+^ erythrocytes to reconstitute whole blood at a 40% haematocrit. Additionally, 200 µL of tail blood was drawn from naïve BALB/c mice and deposited in 20 µL of 1 mg/mL heparin in PBS. Blood was centrifuged at 5000×*g* for 4.5 min to isolate plasma. The RBCs were washed one time with 1 mL of PBS. The mouse plasma was then spiked with the same concentrations of rPfHRP2 and added to the washed RBCs to reconstitute whole blood at 40% haematocrit. Plasma/sera samples containing no PfHRP2 protein were used as controls. All were incubated at 37 °C for at least 12 h. After incubation, samples were centrifuged to separate plasma from RBCs. RBCs were subsequently washed 4 times with 1 mL of PBS. Three individual replicates were performed.

### In vivo model for measuring recombinant PfHPR2 plasma kinetics

Naïve, 8-week old female BALB/c mice received an intraperitoneal injection of 20 µg of recombinant PfHRP2 (rPfHRP2) in 200 µL of PBS. Tail blood (50 or 100 µL) was deposited in 1 mg/mL heparin in PBS to prevent clotting. Blood was centrifuged at 5000×*g* for 4.5 min to isolate plasma. The remaining RBCs were washed two times with 1 mL of PBS.

### Data analysis

The majority of data was analysed using GraphPad Prism (v.5). Data are graphed as mean ± SEM. PfHRP2 levels in the RBCs and plasma were extrapolated using a standard curve and linear regression analysis. Protein half-life was determined using a first-order decay equation. For the comparison between splenic and asplenic mice, data were normalized to percent of total remaining. The statistical methods used include: the Wilcoxon signed-rank test or Mann–Whitney U test to measure differences in the means of two matched or unmatched groups, first-order decay analysis to calculate half-life, log-rank survival analysis to compare clearance times, and a random-effects GLS regression model for analysing associations between tgPfHRP2 in the plasma and RBC fractions of splenic or asplenic mice. The random-effects GLS regression was used to account and adjust for within-subject correlation, using random intercept and robust standard errors. This analysis was performed with Stata version 14 (StataCorp LP, College Station, USA).

## Results

### Persistence of PfHRP2 within human RBCs

**Fig. 1:**
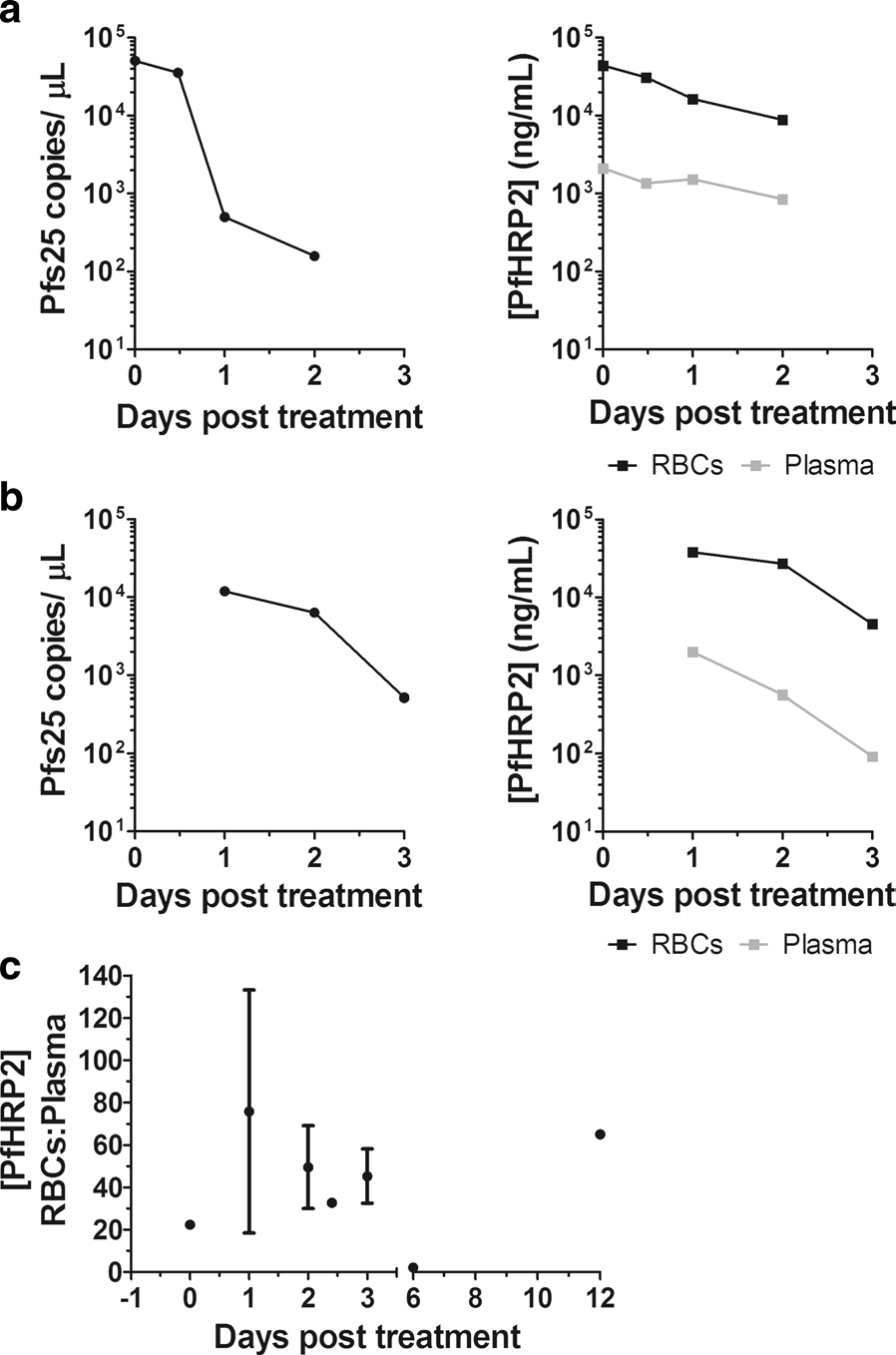
Kinetics of PfHRP2 following *P. falciparum* infection. **a**, **b** Blood was drawn from patients and fractionated into plasma and RBCs. Parasite genomic DNA clearance in a representative single patient post-treatment with artemether–lumefantrine (**a**, left) or atovaquone–proguanil (**b**, left) was quantified using TaqMan primers and probes specific for *P. falciparum* Pfs25. PfHRP2 protein levels were quantified in the plasma and RBCs post-treatment with artemether–lumefantrine (**a**, right) and atovaquone–proguanil (**b**, right) by ELISA. **c** The ratio of PfHRP2 concentration in the RBCs to plasma in each patient (n = 6) was calculated over the days post-treatment. Data are represented as mean ± SEM

**Fig. 2:**
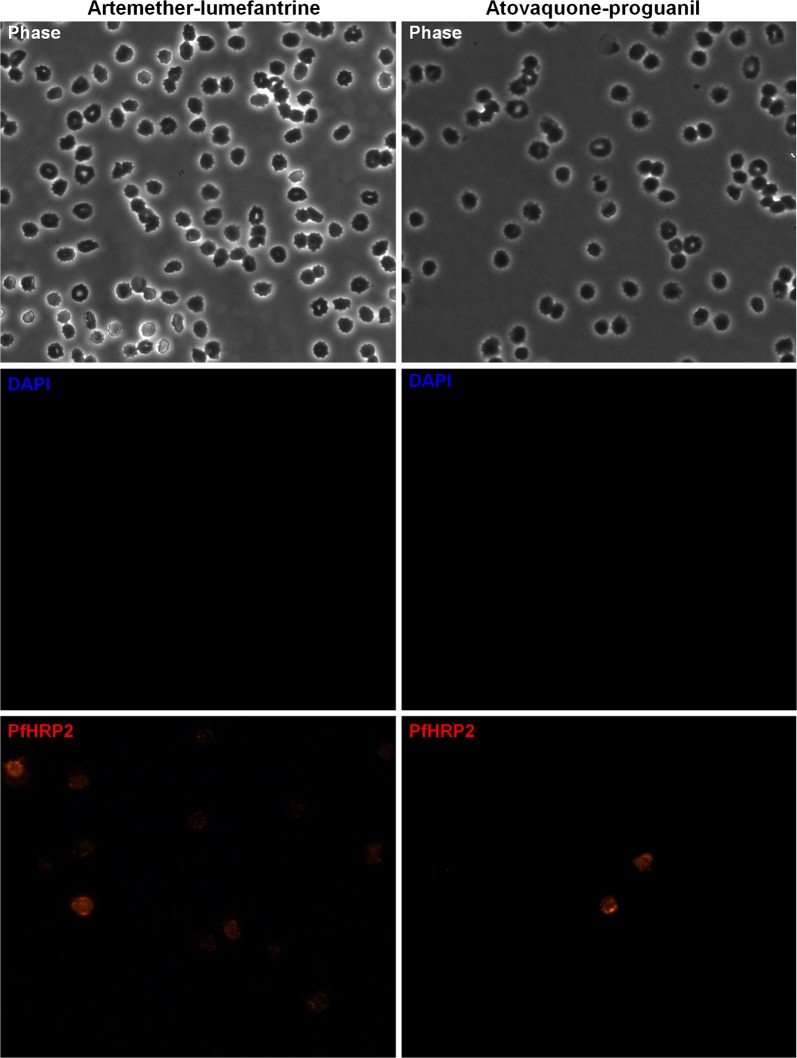
Presence of once-infected RBCs post-treatment with different anti-malarials. Once-infected RBCs were detected in patients on day 3 post-treatment with either artemether–lumefantrine (left) or atovaquone–proguanil (right). Once-infected RBCs were defined as cells with positive PfHRP2 staining (red) but no DAPI staining (blue)

The persistence of PfHRP2 in whole blood has been documented [10–13], but has not been extensively characterized in the blood’s individual compartments, the plasma and the RBCs. We obtained whole blood from 6 patients diagnosed with *P. falciparum* malaria at the hospital laboratory with parasitaemias ranging from < 0.5 to 12%, as measured by microscopy (Table 1). The whole blood was fractionated into plasma and washed RBCs for a quantitative, compartmental analysis of PfHRP2 by ELISA before and after treatment with anti-malarial combinations of artemether–lumefantrine (n = 2) or atovaquone–proguanil (n = 4) (Table 1; Fig. 1a, b). The initial ratio of RBC to plasma levels of PfHRP2 prior to treatment approximated 20-fold and this ratio tended to increase to 40-fold or greater following treatment with both anti-malarial regimens (Table 1; Fig. 1c). PfHRP2 levels in the RBC fraction only declined three-to tenfold in the days following either treatment regimen, while genomic copies of the *P. falciparum* Pfs25 gene decreased 20- to 100-fold (Table 1; Fig. 1a, b). All iRBCs cleared from the peripheral blood within 2–3 days post-treatment when measured by microscopy, despite parasite genomic DNA remaining detectable when measured by qPCR (Table 1). To identify the source of PfHRP2 in the blood post-parasite clearance, we performed immunofluorescence microscopy on RBCs with an antibody specific for PfHRP2 and DAPI staining to identify parasites. Remarkably, we observed PfHRP2 positive, but DNA negative, RBCs at approximately 0.1–1% of total RBCs post-treatment, specifically from days 1 to 3 and day 12 post-treatment (Table 1; Fig. 2; Additional file 3). This indicated the presence of what was likely once-infected RBCs, in which the parasite was cleared, but PfHRP2 remains within the cytoplasm of the RBC. The phenomenon was observed after treatment with both artemether–lumefantrine and atovaquone–proguanil in the small number of patients we examined (Table 1; Fig. 2). Also, in the 6 patients tested, no once-infected RBCs were observed pre-treatment (Additional file 4).

**Table 1:** Quantification of RBCs positive for PfHRP2 but parasite negative in patients post-treatment for *P. falciparum*

Pt	Days post-treatment	Pfs25 copies/µL	RBC PfHRP2 (ng/mL)	Plasma PfHRP2 (ng/mL)	PfHRP2 RBC/Plasma ratio	% of iRBCs/total RBCs	% of PfHRP2 positive/total RBCs	Treatment
1	1	482,693	183,208	7116	26	11.6	Not done	Doxycycline, quinidine, artemether–lumefantrine
	2	2759	160,177	2289	70	< 0.5	3.7	
	2.4	502	74,179	2272	33	0	5.2	
	3	150	79,094	1214	65	0	6.5	
	12	0	25,830	397	65	0	3.5	
2	1	11,945	37,948	1999	19	1.2	Not done	Atovaquone–proguanil
	3	516	4535	92	49	< 0.5	0.38	
3	0	50,398	43,787	2113	21	2.8	0	Artemether–lumefantrine
	1	498	16,370	1529	11	0	1.2	
	2	158	8821	847	10	0	0.63	
4	1	14,289	21,140	85	248	1.0	Not done	Quinine, atovaquone–proguanil
	6	0	43	21	2	0	1.7	
5	0	24	277	11	25	< 0.5	Not done	Atovaquone–proguanil
	1	12,863	38,051	3	12,683	1.1	Not done	
	3	101	2759	129	21	0	0	
6	2	3141	12,372	181	68	< 0.5	0.13	Atovaquone–proguanil

*Pt* patient

### Transgenic PbPfHRP2 parasites synthesize and export the PfHRP2 protein

**Fig. 3:**
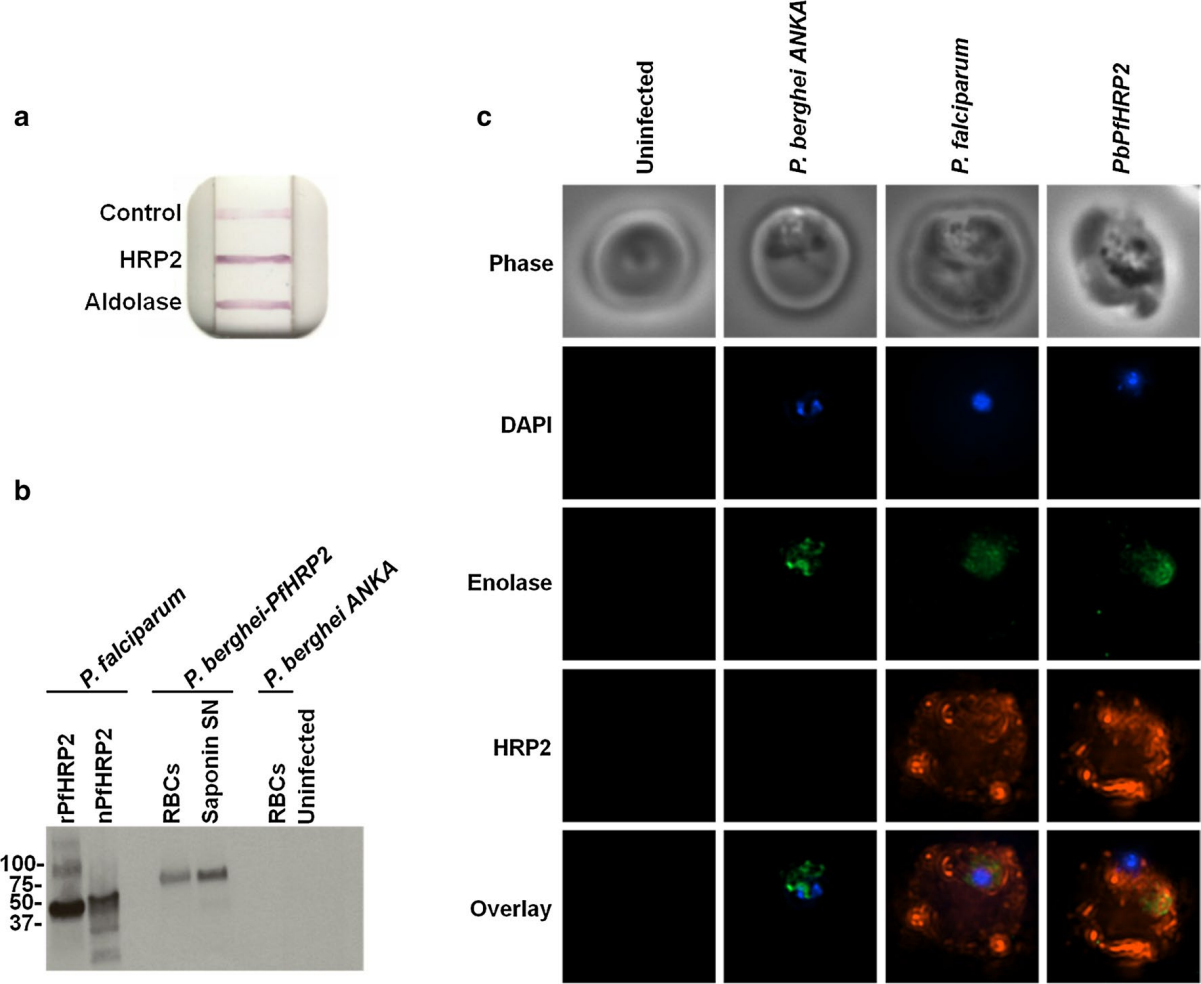
Characterization of the synthesis and localization of PfHRP2 by transgenic PbPfHRP2 parasite. **a** BinaxNOW Malaria RDT™ detecting tgPfHRP2 and *Plasmodium* aldolase in the whole blood of a PbPfHRP2 infected mouse. **b** tgPfHRP2 was detected in iRBCs, and exported to the iRBC cytoplasm (Saponin, SN). RBCs were lysed with saponin to isolate their cytoplasmic fraction. Purified recombinant and native PfHRP2 protein were run as controls. **c** tgPfHRP2 (red) localized within the parasite, demarcated by *Plasmodium* enolase staining (green), and the RBC cytosol, similar to what was observed in *P. falciparum* iRBCs. An uninfected mouse RBC and a *P. berghei* ANKA iRBC that does not produce PfHRP2 are shown as controls

In order to fine tune the dynamics of PfHRP2 in vivo, a controlled experimental model was utilized in which a *P. berghei* rodent parasite (PbPfHRP2), which lacks the PfHRP2 gene family, was engineered to express PfHRP2 (tgPfHRP2) (Additional file 2). Expression and localization of the transgenic PfHRP2 protein (tgPfHRP2) was validated in the novel mouse model by western blot and immunofluorescence (Fig. 3). The presence of tgPfHRP2 in the whole blood of BALB/c mice was detected using a BinaxNOW Malaria Rapid Diagnostic Test™ that qualitatively detects PfHRP2 and pan-species aldolase (Fig. 3a). Western blot analysis confirmed the presence of tgPfHRP2 in the iRBCs (Fig. 3b). tgPfHRP2 was detected in the RBC cytosolic fraction after saponin lysis (Fig. 3b), suggesting it was exported into the iRBC cytoplasm. Immunofluorescence microscopy on PbPfHRP2 iRBCs was performed using monoclonal antibodies against PfHRP2 and *Plasmodium* enolase, a known parasite-localized protein, to differentiate the parasite from the iRBC cytoplasm. In this assay, tgPfHRP2 localized to both the parasite and iRBC cytoplasm (Fig. 3c). Importantly, the PfHRP2 staining observed in PbPfHRP2 iRBCs mimicked the staining of *P. falciparum* iRBCs (Fig. 3c). PfHRP2 was not detected in negative controls, including RBCs infected with the *P. berghei* ANKA wild-type strain that does not express PfHRP2 and uninfected RBCs (Fig. 3b, c).

### Investigating kinetics of tgPfHRP2 during infection with PbPfHRP2

**Fig. 4:**
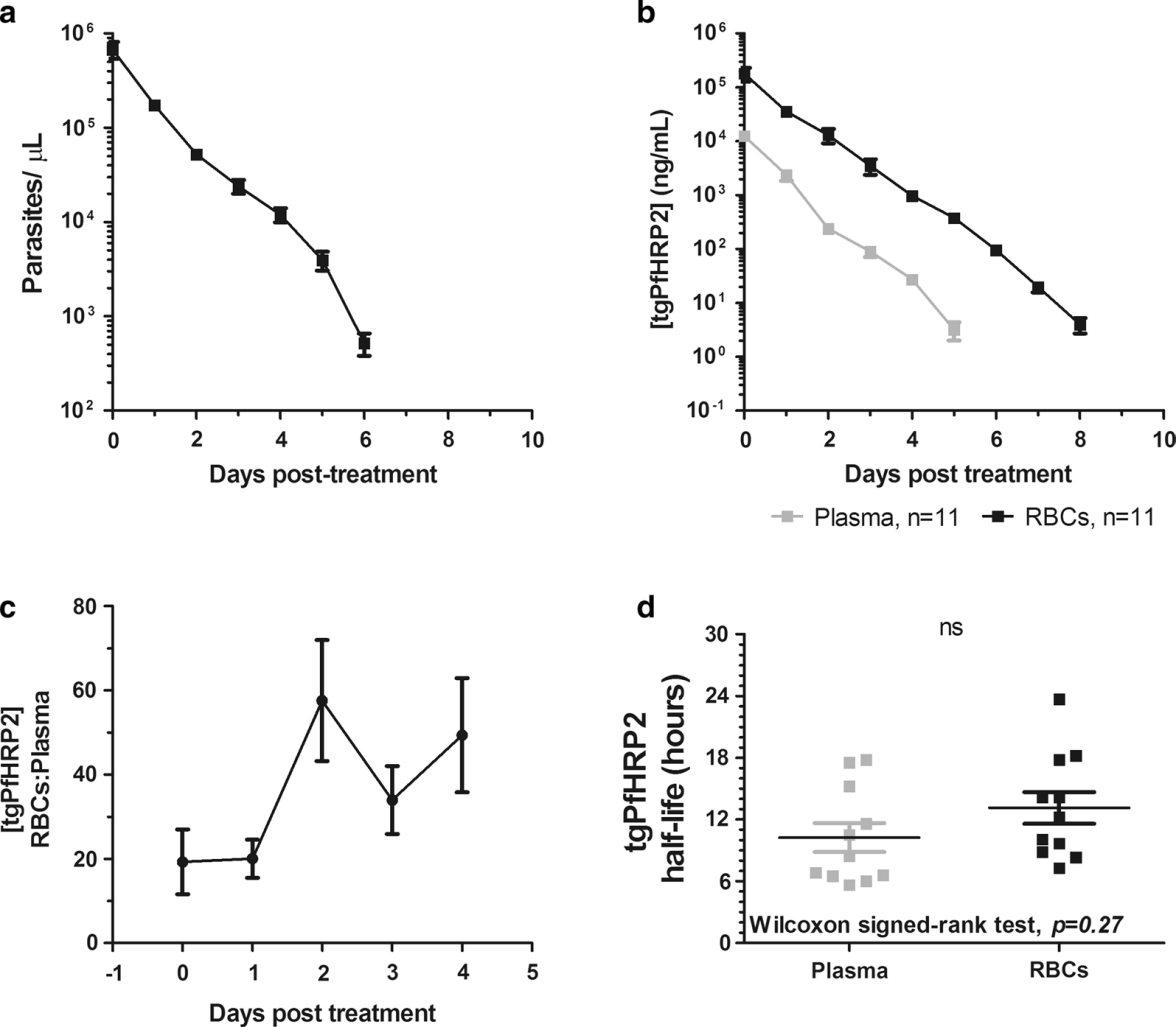
Kinetics of tgPfHRP2 following PbPfHRP2 infection. Tail blood was extracted daily from PbPfHRP2 infected female BALB/c mice post-treatment with artesunate and pyronaridine administered for cure (n = 11). Blood was fractionated into plasma and RBCs. **a** Parasite genomic DNA clearance post-treatment was quantified using qPCR, with TaqMan primers and probes specific for *P. berghei* ANKA 18S rRNA. Data are represented as mean ± SEM. **b** tgPfHRP2 protein levels post-treatment were quantified in the two blood fractions by ELISA. Data are represented as mean ± SEM. **c** The ratio of tgPfHRP2 concentration in the RBCs to plasma in each mouse was calculated over the first 4 days post-treatment. Data are represented as mean ± SEM. **d** The half-life of the tgPfHRP2 was quantified in the plasma and washed RBCs using a first-order decay equation model constrained at a plateau of Y = 0. Each point represents an individual mouse with mean ± SEM displayed. Data were analysed using a Wilcoxon signed-rank test

BALB/c mice were infected with PbPfHRP2 and 5 days post-infection, reached an average parasitaemia of 10.5%, as measured by microscopy, or 675,283 parasites/µL quantified by qPCR (Fig. 4a). On day 5 post-infection, mice were treated with three doses of 50 mg/kg of artesunate (0–8–24 h post-initial treatment) and 36 mg/kg of pyronaridine (24 h post-initial treatment) to cure mice of parasites (Fig. 4a). At this peak parasitaemia prior to treatment, the average concentrations of tgPfHP2 in the plasma and RBCs were 12,512 ng/mL and 179,433 ng/mL, respectively (Fig. 4a, b). tgPfHRP2 levels were significantly higher in the RBC fraction (10–20 fold) compared to the plasma over the days post-treatment (*p *≤ 0.0001, random-effects GLS regression model) (Fig. 4b, c). The half-life of tgPfHRP2 following treatment was calculated for each blood fraction using a first-order decay equation. While not significantly different, tgPfHRP2 had a slightly longer half-life in the RBCs at 13.1 h (median R^2^ = 0.989) compared to 10.2 h in the plasma (median R^2^ = 0.999) (*p *= 0.27, Wilcoxon signed-rank test) (Fig. 4d).

### Determining tgPfHRP2 persistence duration in the plasma and RBCs compared to microscopy and PCR

**Fig. 5:**
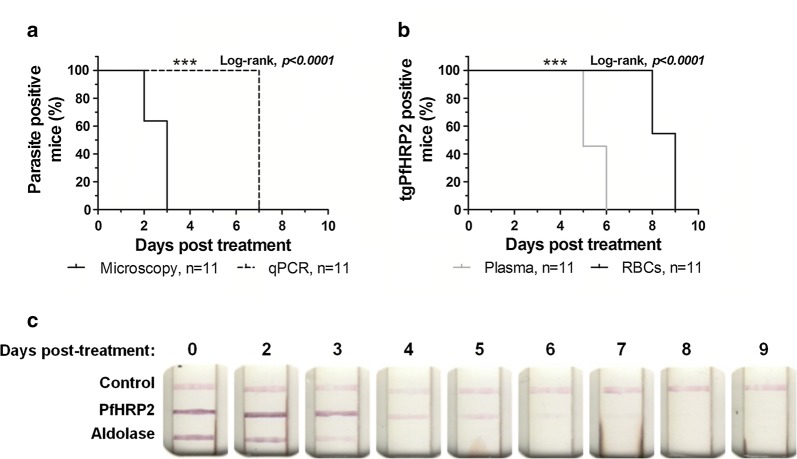
Clearance times of PbPfHRP2 parasites and tgPfHRP2 protein. Female BALB/c mice infected with the PbPfHRP2 parasite (n = 11) were administered artesunate and pyronaridine to cure. **a** Parasite clearance and cure were determined by daily Giemsa-stained blood films and qPCR performed on DNA extracted from whole blood, using TaqMan primers and probes specific for *P. berghei* ANKA 18S rRNA. **b** Blood extracted from each infected mouse was separated into plasma and RBC fractions to measure the presence and clearance of tgPfHRP2 post-treatment by ELISA. Curves were compared using a log-rank test (***p < 0.001; **p < 0.01; *p < 0.05). **c** Representative BinaxNOW Malaria RDTs demonstrating the clearance time of tgPfHRP2 and *Plasmodium* aldolase from the whole blood of a PbPfHRP2 infected mouse that was administered artesunate and pyronaridine to cure

Median parasite clearance time measured by microscopy was 3 days post-treatment (Fig. 5a). However, the median parasite genomic DNA clearance time quantified by qPCR was significantly longer at 7 days (*p * ≤ 0.0001, log-rank) (Fig. 5a). Median plasma tgPfHRP2 clearance time was 5 days while RBC clearance was significantly longer at 9 days (*p* ≤ 0.0001, log-rank) (Fig. 5b). Consequently, tgPfHRP2 positivity in the different blood fractions, particularly within the RBC fraction, outlasted detectable parasites by microscopy (Fig. 5a, b). Parasite genomic DNA was detected by qPCR longer than plasma tgPfHRP2, but protein in the RBCs persisted at least 1-day post genomic DNA clearance (Fig. 5a, b). tgPfHRP2 clearance time from the RBCs measured by ELISA correlated with its clearance time from whole blood of 8–9 days measured by RDT (Fig. 5c). RDT analysis also demonstrated that the *Plasmodium* aldolase protein was eliminated from the whole blood on day 4 post-treatment, shortly after parasites were no longer detected in peripheral blood (Fig. 5c).

### Identifying once-infected, tgPfHRP2 positive murine RBCs post-treatment

**Fig. 6:**
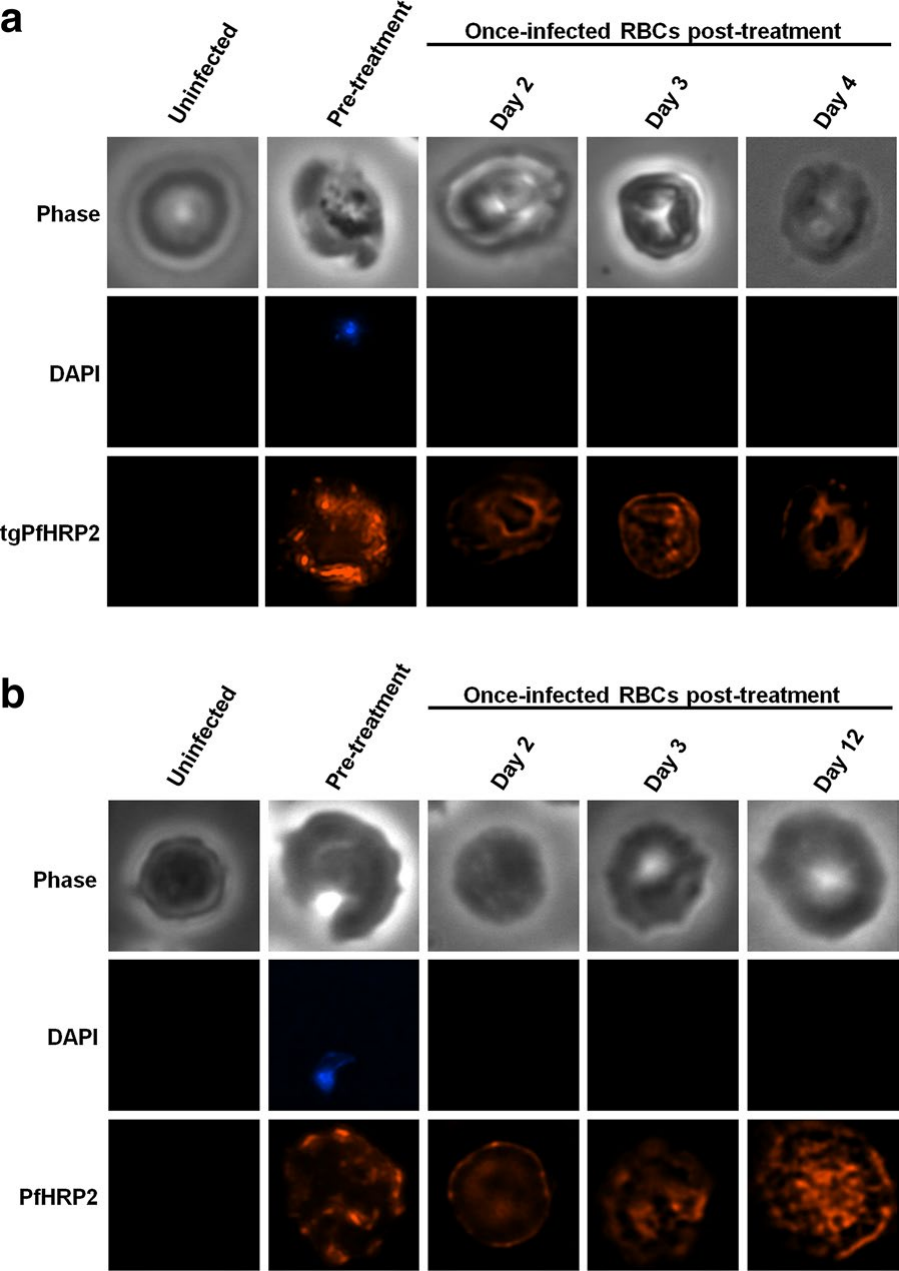
Investigation of PfHRP2 persistence in once-infected RBCs post-treatment. **a** PbPfHRP2 iRBCs were isolated from mice pre-treatment and once-infected RBCs were isolated on days 2, 3, and 4 post-treatment with artesunate and pyronaridine. Uninfected mouse RBCs were used as a control. **b** *P. falciparum* iRBCs were collected from a patient pre-treatment. RBCs once-infected with *P. falciparum* were isolated from the same human patient on days 2, 3, and 12 post-treatment with artemether–lumefantrine. Uninfected human RBCs were used as a control. Once-infected RBCs were defined as cells with positive PfHRP2 staining (red) but no DAPI staining (blue)

The source of tgPfHRP2 in RBCs after parasite clearance was hypothesized to be once-infected RBCs, positive for PfHRP2 but parasite negative, as were observed in human patients post-treatment (Fig. 2). Immunofluorescence microscopy on mouse RBCs was performed with an antibody specific for PfHRP2 and DAPI staining to identify parasites. tgPfHRP2 was detected in iRBCs (DAPI positive) isolated pre-treatment as well as in RBCs isolated 2, 3, and 4 days post-treatment that were DAPI negative (Fig. 6a). This indicates the presence of once-infected RBCs positive for PfHRP2 in their cytoplasm in our mouse model. Importantly, PfHRP2 was not detected in naive mouse or human RBCs, indicating specific recognition of parasite derived PfHRP2 (Fig. 6). PfHRP2-positive, parasite-negative, once-infected RBCs that were observed in a human patient on days 2, 3 and 12 post-treatment with artemether–lumefantrine for a *P. falciparum* infection with an 11.6% initial parasitaemia (Fig. 6b), were morphologically similar to those identified in the mouse model (Fig. 6).

### Investigating potential binding of PfHRP2 to uninfected, naïve RBCs

Slower clearance of PfHRP2 from the RBC fraction in humans and mice could be the result of plasma PfHRP2 binding to naïve RBCs in circulation. To investigate binding, partitioning of PfHRP2 was measured between naïve RBCs and plasma isolated from *P. falciparum* infected patients or human serum/mouse plasma spiked with recombinant PfHRP2 at average levels > 100,000 ng/mL or < 30,000 ng/mL as measured by ELISA, that were incubated together in vitro (Additional file 5: Table S2). Binding was defined as absorbance levels above those measured for RBCs incubated with naïve plasma. While protein levels quantified by ELISA were highly variable in the RBC and plasma fractions within and across replicates, the average percent of total PfHRP2 protein bound to uninfected, naïve mouse or human RBCs in vitro was extremely low, measuring below 0.5% for all plasma/serum samples tested (Additional file 5: Table S2).

Next, binding was investigated in vivo. BALB/c mice (n = 3) were injected with 100,000 ng/mL of recombinant PfHRP2 (rPfHRP2) in order to achieve high plasma protein levels and mimic plasma protein circulation and elimination observed during severe malaria infections (> 10% parasitaemia) [20, 27, 38]. ELISA was used to quantify concentration and partitioning of rPfHRP2 in the RBC and plasma fractions isolated from mice at regular intervals post-inoculation (Additional file 6: Table S3, Additional file 7). At 0.5 h post-inoculation, the average concentration of rPfHRP2 quantified in the plasma was 35,113 ng/mL (Additional file 7).

Minimal binding to uninfected RBCs was detected within the first 12 h post-inoculation, on the order of 3–13 ng/mL compared to the 900–35,000 ng/mL of rPfHRP2 in the plasma at those same time points (Additional file 6: Table S3). Consequently, the ratios of rPfHRP2 in the uninfected RBCs compared to the plasma in the first 12 h were extremely low, averaging 0.004 (Additional file 6: Table S3). The percent of the total protein (RBC + plasma) that bound to the RBCs over the time course was ≤ 1% (Additional file 6: Table S3). No binding was detected in the naïve RBCs after 24 h, despite plasma protein remaining detectable for a median of 4 days post-inoculation (Additional file 7; Additional file 6: Table S3). rPfHRP2 plasma clearance followed a first-order decay process, with a very short calculated plasma half-life of 1.9 h (median R^2^ = 0.998) (Additional file 7).

### Comparing parasite and tgPfHRP2 clearance in splenic and asplenic mice

**Fig. 7:**
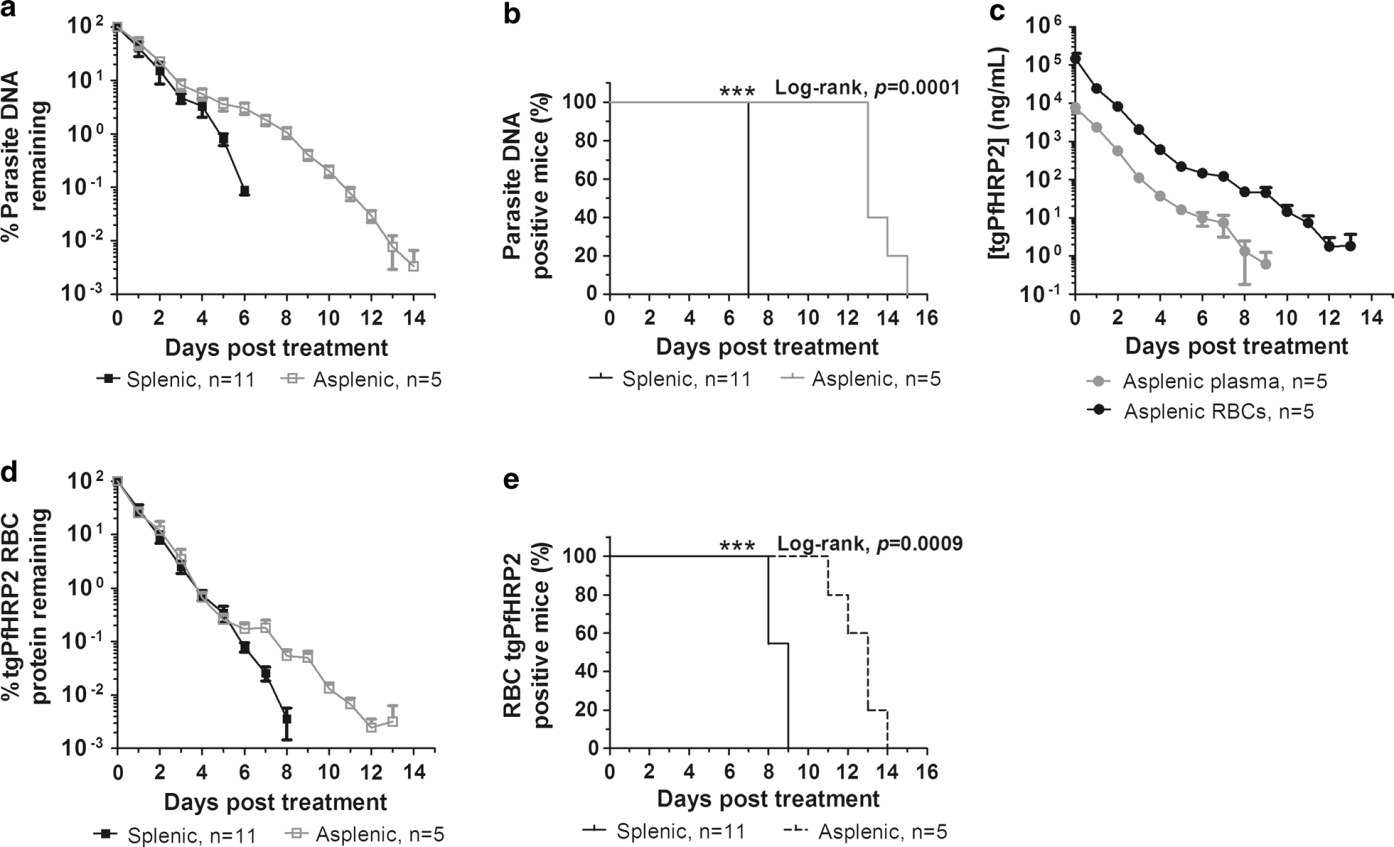
tgPfHRP2 protein kinetics following infection in splenectomized mice. Female BALB/c mice (n = 5) were splenectomized and infected with the PbPfHRP2 parasite. Artesunate and pyronaridine were administered for cure. **a**, **b** Parasite genomic DNA clearance post-treatment was quantified in the splenectomized mice using qPCR, with TaqMan primers and probes specific for *P. berghei* ANKA 18S rRNA, and compared to parasite clearance time in mice with intact spleens (n = 7). Data were normalized by calculating percent of total parasite DNA remaining (**a**). Overall clearance time for parasite DNA was compared in splenic and asplenic mice using a log-rank test (***p < 0.001; **p < 0.01, *p < 0.01) (**b**). Data are represented as mean ± SEM. **c** Tail blood was extracted daily from splenectomized mice to quantify the concentration of tgPfHRP2 post-treatment in the plasma and RBCs by ELISA. Data are represented as mean ± SEM. tgPfHRP2 levels post-treatment in the RBCs of both the splenic and asplenic mice were compared. Data were normalized by calculating percent of total tgPfHRP2 remaining (**d**). Overall clearance time for RBC tgPfHRP2 was compared in splenic and asplenic mice using a log-rank test (***p < 0.001; **p < 0.01, *p < 0.01) (**e**). Data are represented as mean ± SEM

Because RBC pitting occurs in the spleen, how splenectomy in mice alters clearance, persistence, and kinetics of tgPfHRP2 following infection with the PbPfHRP2 parasite and treatment with artesunate and pyronaridine was investigated. The parasitaemia prior to treatment (4–5 days post-infection) determined by qPCR was significantly different between the splenic (675,283 parasites/µL) and asplenic (1,429,170 parasites/µL) mice (Mann–Whitney U test, *p *= 0.041). To normalize the data, the percent of total parasite DNA remaining was calculated (Fig. 7a). On average, 71% of total parasite DNA cleared on day 1 post-treatment in splenic mice, but only 49% cleared in asplenic mice (Fig. 7a). Additionally, in mice with intact spleens, all parasite DNA cleared by day 7 post-treatment, but in asplenic mice, DNA was detectable at very low levels (≤ 1%) from days 12 to 14 post-treatment, leading to a significantly longer overall clearance time of 13 days (*p *= 0.0001, log-rank) (Fig. 7a, b). When measuring parasite clearance by microscopy, no differences between splenic and asplenic mice were observed, as both demonstrated a median clearance time of 3 days.

In asplenic mice, the concentration of tgPfHRP2 was significantly higher in the RBCs compared to the plasma over the days post-treatment (*p *= 0.005, random-effects GLS regression model) (Fig. 7c), which was the same trend observed in splenic mice. tgPfHRP2 elimination from the different blood fractions post-treatment also followed a first-order decay process in the mice lacking spleens. In the asplenic mice, the average tgPfHRP2 plasma half-life was 16.4 h (median R^2^ = 0.990) and the average half-life in the RBCs was not significantly different at 13.4 h (median R^2^ = 0.998) (*p *= 1.00, Wilcoxon signed-rank test). The starting tgPfHRP2 concentration in the RBC fraction of PbPfHRP2 infected mice prior to treatment was higher in the splenic group (179,432.6 ng/mL) compared to the asplenic group (144,806 ng/mL), despite a lower parasite load calculated by qPCR. Data was normalized to percent of total tgPfHRP2 remaining in the RBCs (Fig. 7d). Interestingly, when comparing percent of total protein remaining in the RBCs post-treatment for the splenic and asplenic mice, the initial tgPfHRP2 clearance rate for more than 99% of the protein was not different within the first 6 days post-treatment (Fig. 7d). Consequently, the tgPfHRP2 RBC elimination half-lives in splenic (13.1 h) and asplenic mice (13.4 h) were not significantly different (*p *= 1.00, Mann–Whitney U Test). However, lingering, low levels of tgPfHRP2 (< 1%) were detected in the RBCs of the asplenic mice during days 10–14 post-treatment, leading to a significantly longer overall median clearance time of 13 days (*p *= 0.0009, log-rank) (Fig. 7d, e). This was not observed in the splenic group, which had a median clearance time of 9 days. As in splenic mice, tgPfHRP2 persisted significantly longer in the RBCs (11–14 days) than the plasma (6–10 days) in asplenic mice (Fig. 7d). One apparent difference between these groups was that in asplenic mice, the tgPfHRP2 protein in the RBCs cleared at the same time or before parasite genomic DNA (Fig. 7b, e). In the splenic mice, at least a 1-day difference in clearance time was observed (Fig. 5).

## Discussion

Because PfHRP2 is expressed solely by *P. falciparum*, previous experimentation to measure PfHRP2 kinetic parameters have relied on in vitro culture and human studies using mostly whole blood and occasionally plasma [8–10, 39]. Here the compartmentalization of PfHRP2 in the blood was explored in limited human clinical samples and correlated some of the findings in a novel transgenic *P. berghei* parasite (PbPfHRP2) engineered to express PfHRP2 under the control of the HSP70 promoter. Consistently higher levels of PfHRP2 in the RBC fraction compared to the plasma was demonstrated over the days post-treatment in both humans and the transgenic PbPfHRP2 mouse model, which is consistent with data that previously demonstrated lower plasma PfHRP2 levels compared to overall levels in the whole blood post-treatment [10].

There is recent evidence that persistence of the PfHRP2 antigen is due to the clearance of dying and non-viable parasites by the process of erythrocyte pitting in the spleen, in which the parasite is removed by splenic macrophages from an intact RBC that then returns to circulation as a once-infected RBC [10, 40]. These once-infected RBCs have been identified and quantified previously by the presence of the *P. falciparum* ring-infected erythrocyte surface antigen (RESA) using flow cytometry and immunofluorescence microscopy [10, 41]. RESA, like PfHRP2, is exported into the cytoplasm of the iRBC and associates with the iRBC membrane [42]. Recently, Ndour et al. also identified PfHRP2 in these once-infected RBCs isolated from *P. falciparum* patients 3 days post-artesunate treatment [10]. If PfHRP2 is persisting within circulating once-infected RBCs, this would predict slower protein clearance from the RBC fraction of the blood compared to the plasma. This was demonstrated in human patients treated with either artemether–lumefantrine or atovaquone–proguanil. While the number of patients investigated was small, it appears that this pitting phenomenon potentially not only results from artesunate treatment and should be further investigated in regards to treatment with other anti-malarial regimens. In the transgenic mouse model post-treatment with artesunate and pyronaridine, tgPfHRP2 also cleared more slowly from the RBCs than plasma. Once-infected, PfHRP2 positive, parasite negative RBCs were abundantly detected post-treatment in humans (at 0.1–1%) and were also observed in our mouse model. The results confirm previous observations [10] and suggest that these once-infected RBCs that remain PfHRP2 positive and circulate in the peripheral blood could be contributing to the long duration of RDT positivity despite the lack of an active infection. In the mouse model, tgPfHRP2 persisted in the RBCs for at least 5–7 days beyond measurable peripheral blood parasitaemia by microscopy. However, tgPfHRP2 only persisted 1 day post parasite genomic DNA clearance time as measured by qPCR for the *P. berghei ANKA* 18S rRNA.

Because erythrocyte pitting is a method of parasite removal in the spleen, the impact of splenectomy on the in vivo tgPfHRP2 kinetics in the RBCs was investigated. The spleen plays a central role in clearing parasites during infections with *P. falciparum* [43–45] and lethal rodent malaria strains [46, 47]. It has been demonstrated that in asplenic patients post-artesunate, treatment, parasite clearance was prolonged, though most parasites appeared dead and were unable to be cultured ex vivo [43, 48]. Consequently, the spleen is necessary for parasite clearance following the rapid anti-malarial activity of artemisinin-based compounds [48]. In the transgenic mouse model, we observed lingering, low levels of parasite DNA in the asplenic mice, leading to a longer overall median clearance time of 13 days. This could suggest that parasite DNA may persist after parasites are killed.

Supporting the hypothesis that erythrocyte pitting depends on the spleen, once-infected, RESA positive RBCs have not been observed in splenectomized patients [43], nor were they observed in vitro, following artesunate treatment of *P. falciparum* culture [49]. While the clearance of more than 99% of the tgPfHRP2 protein from the RBC fraction was similar in splenic and asplenic mice, there was a slower clearance of the remaining protein in asplenic mice. Parasite clearance mechanisms in the absence of a spleen have not been extensively characterized, particularly in mice, but the liver along with other lymphoid tissues may play a role [46]. One potential explanation could be that without the spleen to clear parasites, slower parasite clearance overall could result in prolonged detection of tgPfHRP2 in RBCs.

Additionally, PfHRP2 could be binding uninfected RBCs in circulation upon its release from iRBCs, contributing to persistence. Little binding of PfHRP2 to naïve RBCs was observed, as the percent of total protein bound was under 1–0.5% for all conditions tested. Transmission electron microscopy images and protein localization analysis from a previous study demonstrated that PfHRP2 was localizing within the cytoplasm, immediately under the plasma membrane of *P. falciparum* once-infected RBCs, further supporting the results [10]. Therefore, it seems binding of PfHRP2 to the surface of uninfected RBCs is negligible and consequently not contributing to persistent antigenaemia.

One important difference observed between our novel transgenic murine model and *P. falciparum* infection in humans is the duration of PfHRP2 persistence after treatment. In mice infected with the transgenic PbPfHRP2, we measured persistent antigenaemia for only 8–9 days following treatment, where PfHRP2 has been detected up to 28–40 days following *P. falciparum* infections [11–13] and once-infected, PfHRP2 positive RBCs were observed as long as 12 days post-treatment in our small human study. These pitted, once-infected *P. falciparum* RBCs have a shorter lifespan (7–28 days) than a naïve human RBC (100–120 days) [10, 41]. Naïve murine RBCs only have a lifespan of approximately 30–40 days [50, 51]. As a result, pitted murine once-infected PbPfHRP2 RBCs could have a shorter lifespan of only a few days, causing shorter persistence duration. Additionally, shorter persistence duration could be due in part to the increased pliability of *P. berghei* iRBCs [52], as erythrocyte pitting seems to correlate with the increased rigidity of *P. falciparum* iRBCs [53]. Also unlike *P. falciparum, P. berghei* preferentially infects reticulocytes [54], which differ from mature RBCs and may not undergo pitting. The splenic architecture of mice and humans differs [55–57], but the ability of murine spleens to mechanically trap RBCs with foreign/inclusion bodies and remove them through a pitting-like mechanism has been demonstrated previously [58]. In fact, the number of Howell-Jolly inclusion bodies increased from 2 to 3% in the peripheral blood of mice following splenectomy [59]. Therefore, it is possible that the trapping and pitting mechanisms employed in the mouse spleen are different from those in humans and may be less sophisticated, leading to less pitting and a shorter duration of PfHRP2 persistence in mice compared to humans. This could also explain the similar kinetics observed for tgPfHRP2 post-treatment in the RBC fractions of splenic and asplenic mice.

## Conclusions

PfHRP2 persistence after treatment presents a critical challenge to diagnostic interpretation of the malaria RDT, but the mechanism underlying this phenomenon has not been extensively characterized. In this study, a transgenic *P. berghei* parasite that expresses PfHRP2 was generated and tgPfHRP2 kinetics was investigated in the plasma and RBC fractions of the whole blood over time post-treatment to correlate with human samples. Persistent PfHRP2 antigenaemia was observed for 8–9 days in mice and appeared to be driven by a slower rate of elimination of PfHRP2 from the RBCs compared to plasma. While the longevity of PfHRP2 antigenaemia in the transgenic mouse model was considerably shorter than what is measured following a *P. falciparum* infection in humans, once-infected RBCs that were positive for the PfHRP2 antigen were detected in mice. The fraction of these once-infected RBCs present over the days post-treatment was quantifiable in a small number of human patients and detected these once-infected RBCs post-treatment with artemether–lumefantrine and atovaquone–proguanil. While only 6 patients were investigated with most timepoints within a few days, this data could indicate that pitting may not solely be driven by artesunate-based anti-malarial regimens but could result from treatment with a variety of anti-malarials, and thus should be further explored. With the limited human samples included in this study, the PfHRP2 half-life in humans was not estimated. It is important to note that time to clearance of half of PfHRP2 is different than half of total time to clear PfHRP2. Overall, these data suggest that erythrocyte pitting in the spleen could be driving persistent protein in the RBCs and these once-infected RBCs are contributing to a longer duration of RDT positivity for PfHRP2 following a successfully treated *P. falciparum* infection.

## Additional files

**Additional file 1: Table S1.** Sequences of primers and fluorescent probes used to quantify PbPfHRP2 and *P. falciparum* infections.

**Additional file 2: Figure S1.** PCR verification that transgenic PbPfHRP2 parasite carries the HRP2 gene. (A) PCR of 5′ and 3′ ends of the HRP2-2A-GFP transgene from genomic DNA purified from whole blood from wild-type *P. berghei* ANKA (lane 1) or PbPfHRP2 infected Swiss Webster mice (lane 2). (B) Integration PCR of genomic DNA purified from whole blood of wild-type *P. berghei* ANKA (lane 1 and 3) or PbPfHRP2 infected Swiss Webster mice (lane 2 and 4). Lanes 1 and 2 show amplification upstream of the 5′ integration site of 230p (forward) and the end of plasmid GFP (reverse). Lanes 3 and 4 show amplification at the start of the HRP2 gene (forward) and after the 3′ integration site of 230p (reverse).

**Additional file 3: Figure S2.** Presence of once-infected RBCs post-treatment with different anti-malarials. Once-infected RBCs were detected in a patient that was qPCR negative for Pfs25 on day 12 post-treatment with artemether–lumefantrine (left) and another patient that was qPCR negative for Pfs25 on day 6 post-treatment with atovaquone–proguanil (right). Once-infected RBCs were defined as cells with positive PfHRP2 staining (red) but no DAPI staining.

**Additional file 4: Figure S3.** Investigation of the presence of once-infected RBCs pre-treatment. iRBCs were isolated from a *P. falciparum* infected patient with a parasitaemia of 2.9% quantified by blood film prior to receiving treatment. No once-infected RBCs were observed prior to treatment. iRBCs were defined as cells with positive PfHRP2 (red) and DAPI staining (blue). Once-infected RBCs were defined as cells with positive PfHRP2 staining (red) but no DAPI staining (blue).

**Additional file 5: Table S2.** Distribution of PfHRP2 between naïve human or mouse RBCs and PfHRP2-containing plasma/sera incubated in vitro.

**Additional file 6: Table S3.** Distribution of rPfHRP2 between RBC and plasma fractions isolated from mice (n = 3) post-recombinant protein injection.

**Additional file 7: Figure S4.** Kinetics of recombinant PfHRP2 in plasma following protein injection. Female BALB/c mice (n = 3) were injected with rPfHRP2 protein. Plasma was collected at regular intervals post injection for quantification of rPfHRP2 plasma concentration. The protein half-life in each mouse was calculated and averaged using a first-order decay equation model constrained at a plateau of Y = 0. Data are represented as mean ± SEM.

## Abbreviations

PfHRP2: *P. falciparum* histidine-rich protein II
RDT: rapid diagnostic test
RBC: red blood cell
iRBC: infected red blood cell
PbPfHRP2: transgenic *P. berghei* parasite engineered to express *P. falciparum* histidine-rich protein II
rPfHRP2: recombinant *P. falciparum* histidine-rich protein II
tgPfHRP2: transgenic *P. falciparum* histidine-rich protein II

## References

[1] WHO (2018). World Malaria Report 2017.

[2] Shiff CJ, Premji Z, Minjas JN (1993). The rapid manual *Para*Sight^®^-F test A new diagnostic tool for *Plasmodium falciparum* infection. Trans R Soc Trop Med Hyg..

[3] Beadle C, Long G, McElroy P, Hoffman S, Long G, Weiss W (1994). Diagnosis of malaria by detection of *Plasmodium falciparum* HRP-2 antigen with a rapid dipstick antigen-capture assay. Lancet.

[4] Forney JR, Wongsrichanalai C, Magill AJ, Craig LG, Sirichaisinthop J, Bautista CT (2003). Devices for rapid diagnosis of malaria: evaluation of prototype assays that detect *Plasmodium falciparum* histidine-rich protein 2 and a *Plasmodium vivax*-specific antigen. J Clin Microbiol.

[5] Humar A, Harrington MA, Ohrt C, Pillai D, Kain KC (1997). ParaSight^®^F test compared with the polymerase chain reaction and microscopy for the diagnosis of *Plasmodium falciparum* malaria in travelers. Am J Trop Med Hyg.

[6] Moody A, Hunt-Cooke A, Gabbett E, Chiodini P (2000). Performance of the OptiMAL malaria antigen capture dipstick for malaria diagnosis and treatment monitoring at the Hospital for Tropical Diseases, London. Br J Haematol..

[7] WHO. Guidelines for the treatment of malaria. 3^rd^ Ed. Geneva: World Health Organization; 2015. 26020088

[8] Dondorp AM, Desakorn V, Pongtavornpinyo W, Sahassananda D, Silamut K, Chotivanich K (2005). Estimation of the total parasite biomass in acute falciparum malaria from plasma PfHRP2. PLoS Med..

[9] Plucinski MM, Dimbu PR, Fortes F, Abdulla S, Ahmed S, Gutman J (2018). Posttreatment HRP2 clearance in patients with uncomplicated *Plasmodium falciparum* malaria. J Infect Dis.

[10] Ndour PA, Larréché S, Mouri O, Argy N, Gay F, Roussel C, et al. Measuring the *Plasmodium falciparum* HRP2 protein in blood from artesunate-treated malaria patients predicts post-artesunate delayed hemolysis. Sci Transl Med. 2017;9:eaaf9377. 10.1126/scitranslmed.aaf9377 28679662

[11] Iqbal J, Siddique A, Jameel M, Hira PR (2004). Persistent histidine-rich protein 2, parasite lactate dehydrogenase, and panmalarial antigen reactivity after clearance of *Plasmodium falciparum* monoinfection. J Clin Microbiol.

[12] Mayxay M, Pukrittayakamee S, Chotivanich K, Looaresuwan S, White NJ (2001). Persistence of *Plasmodium falciparum* HRP-2 in succesfully treated acute *falciparum* malaria. Trans R Soc Trop Med Hyg.

[13] Swarthout TD, Counihan H, Senga R, van den Broek I (2007). Paracheck-Pf accuracy and recently treated *Plasmodium falciparum* infections: is there a risk of over-diagnosis?. Malar J..

[14] Karbwang J, Tasanor O, Kanda T, Wattanagoon Y, Ibrahim M, Na-Bangchang K (1996). ParaSight™-F test for the detection of treatment failure in multidrug resistant *Plasmodium falciparum* malaria. Trans R Soc Trop Med Hyg.

[15] Wellems TE, Howard RJ (1986). Homologous genes encode two distinct histidine-rich proteins in a cloned isolate of *Plasmodium falciparum*. Proc Natl Acad Sci USA.

[16] Howard RJ, Uni S, Aikawa M, Aley SB, Leech JH, Lew AM (1986). Secretion of a malarial histidine-rich protein (PfHRP II) from *Plasmodium falciparum*-infected erythrocytes. J Cell Biol.

[17] Sharma YD (1988). Genomic organization, structure and possible function of histidine-rich proteins of malaria parasites. Int J Biochem.

[18] Panton LJ, McPhie P, Lee Maloy W, Wellems TE, Taylor DW, Howard RJ (1989). Purification and partial characterization of an unusual protein of *Plasmodium falciparum*: histidine-rich protein II. Mol Biochem Parasitol.

[19] Parra ME, Evans CB, Taylor DW (1991). Identification of *Plasmodium falciparum* histidine-rich protein 2 in the plasma of humans with malaria. J Clin Microbiol.

[20] Desakorn V, Dondorp AM, Silamut K, Pongtavornpinyo W, Sahassananda D, Chotivanich K (2005). Stage-dependent production and release of histidine-rich protein 2 by *Plasmodium falciparum*. Trans R Soc Trop Med Hyg.

[21] Hayward RE, Sullivan DJ, Day KP (2000). *Plasmodium falciparum*: histidine-rich protein II is expressed during gametocyte development. Exp Parasitol.

[22] Akompong T, Kadekoppala M, Harrison T, Oksman A, Goldberg DE, Fujioka H (2002). Trans expression of a *Plasmodium falciparum* histidine-rich protein II (HRPII) reveals sorting of soluble proteins in the periphery of the host erythrocyte and disrupts transport to the malarial food vacuole. J Biol Chem.

[23] Sullivan DJ, Gluzman I, Goldberg D (1996). *Plasmodium* hemozoin formation mediated by histidine-rich proteins. Science.

[24] Genton B, Paget S, Beck H, Gibson N, Alpers M, Hii J (1998). Diagnosis of *Plasmodium falciparum* infection using ParaSight(R)-F test in blood and urine of Papua New Guinean children. Southeast Asian J Trop Med Public Health.

[25] Mikita K, Thakur K, Anstey NM, Piera K, Pardo C, Weinberg JB (2014). Quantification of *Plasmodium falciparum* histidine-rich protein-2 in cerebral spinal fluid from cerebral malaria patients. Am J Trop Med Hyg.

[26] Wilson NO, Adjei AA, Anderson W, Baidoo S, Stiles JK (2008). Detection of *Plasmodium falciparum* histidine-rich protein II in saliva of malaria patients. Am J Trop Med Hyg.

[27] Desakorn V, Silamut K, Angus B, Sahassananda D, Chotivanich K, Suntharasamai P (1997). Semi-quantitative measurement of *Plasmodium falciparum* antigen PfHRP2 in blood and plasma. Trans R Soc Trop Med Hyg.

[28] Ndonwi M, Burlingame OO, Miller AS, Tollefsen DM, Broze GJ, Goldberg DE (2011). Inhibition of antithrombin by *Plasmodium falciparum* histidine-rich protein II. Blood.

[29] Das P, Grewal JS, Chauhan VS (2006). Interaction of *Plasmodium falciparum* histidine-rich protein II with human lymphocytes leads to suppression of proliferation, IFN-gamma release, and CD69 expression. Parasitol Res.

[30] Pal P, Daniels BP, Oskman A, Diamond MS, Klein RS, Goldberg DE (2016). *Plasmodium falciparum* histidine-rich protein II compromises brain endothelial barriers and may promote cerebral malaria pathogenesis. MBio..

[31] Walker LA, Sullivan DJ (2017). Impact of extended duration of artesunate treatment on parasitological outcome in a cytocidal murine malaria model. Antimicrob Agents Chemother.

[32] Janse CJ, Franke-Fayard B, Mair GR, Ramesar J, Thiel C, Engelmann S (2006). High efficiency transfection of *Plasmodium berghei* facilitates novel selection procedures. Mol Biochem Parasitol.

[33] Waters AP, Thomas AW, van Dijk MR, Janse CJ (1997). Transfection of malaria parasites. Methods.

[34] Dame JB, McCutchan TF. The four ribosomal DNA units of the malaria parasite *Plasmodium berghei*. Identification, restriction map, and copy number analysis. J Biol Chem. 1983;258:6984–90. 6304071

[35] van Spaendonk RM, Ramesar J, van Wigcheren A, Eling W, Beetsma AL, van Gemert GJ (2001). Functional equivalence of structurally distinct ribosomes in the malaria parasite, *Plasmodium berghei*. J Biol Chem..

[36] van Spaendonk RM, Ramesar J, Janse CJ, Waters AP (2000). The rodent malaria parasite *Plasmodium berghei* does not contain a typical O-type small subunit ribosomal RNA gene. Mol Biochem Parasitol.

[37] Noedl H, Yingyuen K, Laoboonchai A, Fukuda M, Sirichaisinthop J, Miller RS (2006). Sensitivity and specificity of an antigen detection ELISA for malaria diagnosis. Am J Trop Med Hyg.

[38] Vácha J (1975). Blood volume in inbred strain BALB/c, CBA/J and C57BL/10 mice determined by means of 59Fe-labelled red cells and 59Fe bound to transferrin. Physiol Bohemoslov..

[39] Marquart L, Butterworth A, McCarthy JS, Gatton ML (2012). Modelling the dynamics of *Plasmodium falciparum* histidine-rich protein 2 in human malaria to better understand malaria rapid diagnostic test performance. Malar J..

[40] Anyona SB, Schrier SL, Gichuki CW, Waitumbi JN, Githeko A, Brandling-Bennett A (2006). Pitting of malaria parasites and spherocyte formation. Malar J..

[41] Newton PN, Chotivanich K, Chierakul W, Ruangveerayuth R, Teerapong P, Silamut K (2001). A comparison of the in vivo kinetics of *Plasmodium falciparum* ring-infected erythrocyte surface antigen-positive and -negative erythrocytes. Blood.

[42] Foley M, Tilley L, Sawyer WH, Anders RF (1991). The ring-infected erythrocyte surface antigen of *Plasmodium falciparum* associates with spectrin in the erythrocyte membrane. Mol Biochem Parasitol.

[43] Chotivanich K, Udomsangpetch R, McGready R, Proux S, Newton P, Pukrittayakamee S (2002). Central role of the spleen in malaria parasite clearance. J Infect Dis.

[44] Quinn TC, Wyler DJ (1980). Resolution of acute malaria (*Plasmodium berghei* in the rat): reversibility and spleen dependence. Am J Trop Med Hyg.

[45] Looareesuwan S, Ho M, Wattanagoon Y, White NJ, Warrell DA, Bunnag D (1987). Dynamic alteration in splenic function during acute *falciparum* malaria. N Engl J Med.

[46] Moore BR, Jago JD, Batty KT (2008). *Plasmodium berghei*: parasite clearance after treatment with dihydroartemisinin in an asplenic murine malaria model. Exp Parasitol.

[47] Weiss L (1989). Mechanisms of splenic control of murine malaria: cellular reactions of the spleen in lethal (strain 17XL) *Plasmodium yoelii* malaria in BALB/c mice, and the consequences of pre-infective splenectomy. Am J Trop Med Hyg.

[48] Thu LTA, Davis TME, Binh TQ, van Phuong N, Anh TK (1997). Delayed parasite clearance in a splenectomized patient with falciparum malaria who was treated with artemisinin derivatives. Clin Infect Dis.

[49] Chotivanich K, Udomsangpetch R, Dondorp A, Williams T, Angus B, Simpson JA (2000). The mechanisms of parasite clearance after antimalarial treatment of *Plasmodium falciparum* malaria. J Infect Dis.

[50] Horky J, Vacha J, Znojil V (1978). Comparison of life span of erythrocytes in some inbred strains of mouse using 14C-labelled glycine. Physiol Bohemoslov..

[51] Magnani M, Rossi L, Stocchi V, Cucchiarini L, Piacentini G, Fornaini G (1988). Effect of age on some properties of mice erythrocytes. Mech Ageing Dev.

[52] Huang S, Undisz A, Diez-Silva M, Bow H, Dao M, Han J (2013). Dynamic deformability of *Plasmodium falciparum*-infected erythrocytes exposed to artesunatein vitro. Integr Biol..

[53] Deplaine G, Safeukui I, Jeddi F, Lacoste F, Brousse V, Perrot S (2011). The sensing of poorly deformable red blood cells by the human spleen can be mimicked in vitro. Blood.

[54] Cromer D, Evans KJ, Schofield L, Davenport MP (2006). Preferential invasion of reticulocytes during late-stage *Plasmodium berghei* infection accounts for reduced circulating reticulocyte levels. Int J Parasitol.

[55] Schmidt EE, MacDonald IC, Groom AC (1985). Microcirculation in mouse spleen (nonsinusal) studied by means of corrosion casts. J Morphol.

[56] Blue J, Weiss L (1981). Vascular pathways in nonsinusal red pulp-an electron microscope study of the cat spleen. Am J Anat..

[57] Steiniger BS (2015). Human spleen microanatomy: why mice do not suffice. Immunology.

[58] Klausner M, Hirsch L, Leblond P, Chamberlain J, Klemperer M, Segel G (1975). Contrasting splenic mechanisms in the blood clearance of red blood cells and colloidal particles. Blood..

[59] Lozzio BB (1972). Hematopoiesis in congenitally asplenic mice. Am J Physiol.

