# Supplementary material for: In vivo compartmental kinetics of Plasmodium falciparum histidine-rich protein II in the blood of humans and in BALB/c mice infected with a transgenic Plasmodium berghei parasite expressing histidine-rich protein II
Source: Malar J. 2019 Mar 13;18:78. doi: 10.1186/s12936-019-2712-3 (PMC6416945; doi:10.1186/s12936-019-2712-3)
Supplement: Supplementary file 1 — Additional file 1: Table S1. Sequences of primers and fluorescent probes used to quantify PbPfHRP2 and P. falciparum infections. [file 12936_2019_2712_MOESM1_ESM.docx]

**Additional Table S1.** Sequences of primers and fluorescent probes used to quantify PbPfHRP2 and *P. falciparum* infections**.**

| **Target** | **Primer/probe** | **Sequence (5'‒3')** |
| --- | --- | --- |
| *P. berghei* 18S rRNA | Forward | GGA GAT TGG TTT TGA CGT TTA TGC G |
|  | Reverse | AAG CAT TAA ATA AAG CGA ATA CAT CCT TA |
|  | Probe | 6-FAM CAA TTG GTT TAC CTT TTG CTC TTT |
| *P. falciparum* Pfs25 | Forward | CCA TGT GGA GAT TTT TCC AAA TGT A |
|  | Reverse | CAT TTA CCG TTA CCA CAA GTT ACA TTC |
|  | Probe | Cy5 CCG TTT CAT ACG CTT GTA A |
