# Supplementary figures and images for: In vivo compartmental kinetics of Plasmodium falciparum histidine-rich protein II in the blood of humans and in BALB/c mice infected with a transgenic Plasmodium berghei parasite expressing histidine-rich protein II
Source: Malar J. 2019 Mar 13;18:78. doi: 10.1186/s12936-019-2712-3 (PMC6416945; doi:10.1186/s12936-019-2712-3)

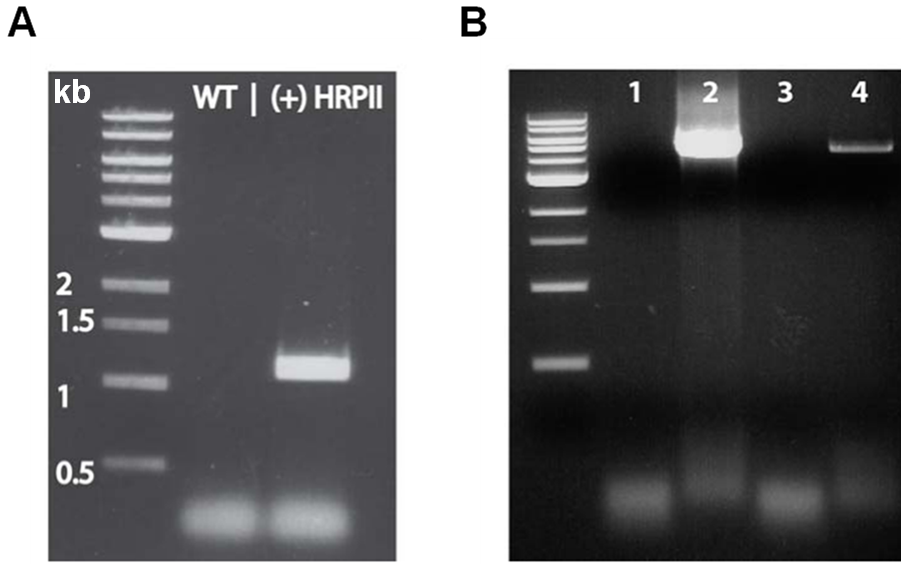

Supplement: Supplementary file 2 — Additional file 2: Figure S1. PCR verification that transgenic PbPfHRP2 parasite carries the HRP2 gene. (A) PCR of 5′ and 3′ ends of the HRP2-2A-GFP transgene from genomic DNA purified from whole blood from wild-type P. berghei ANKA (lane 1) or PbPfHRP2 infected Swiss Webster mice (lane 2). (B) Integration PCR of genomic DNA purified from whole blood of wild-type P. berghei ANKA (lane 1 and 3) or PbPfHRP2 infected Swiss Webster mice (lane 2 and 4). Lanes 1 and 2 show amplification upstream of the 5′ integration site of 230p (forward) and the end of plasmid GFP (reverse). Lanes 3 and 4 show amplification at the start of the HRP2 gene (forward) and after the 3′ integration site of 230p (reverse). [file 12936_2019_2712_MOESM2_ESM.tif]

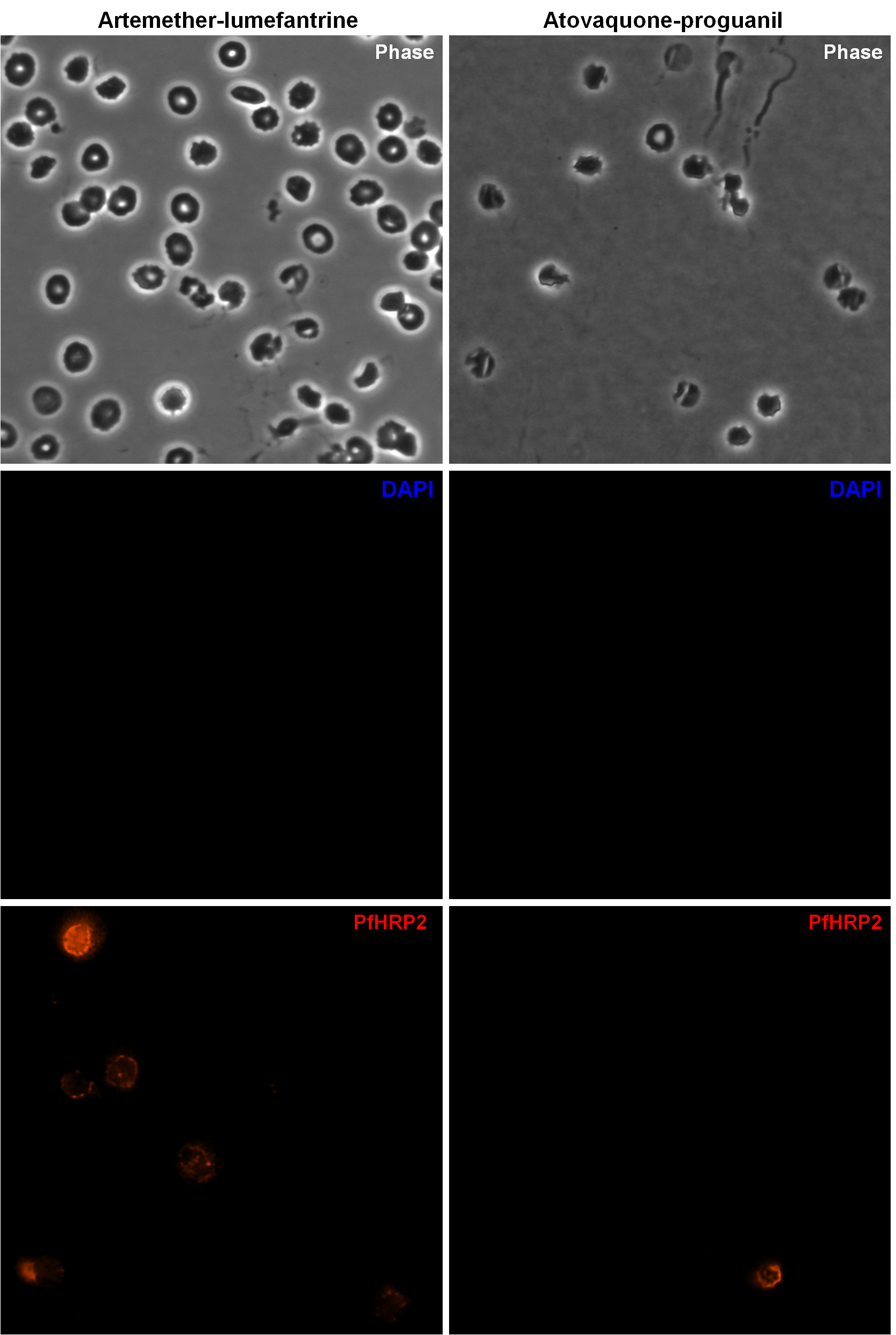

Supplement: Supplementary file 3 — Additional file 3: Figure S2. Presence of once-infected RBCs post-treatment with different anti-malarials. Once-infected RBCs were detected in a patient that was qPCR negative for Pfs25 on day 12 post-treatment with artemether–lumefantrine (left) and another patient that was qPCR negative for Pfs25 on day 6 post-treatment with atovaquone–proguanil (right). Once-infected RBCs were defined as cells with positive PfHRP2 staining (red) but no DAPI staining. [file 12936_2019_2712_MOESM3_ESM.tif]

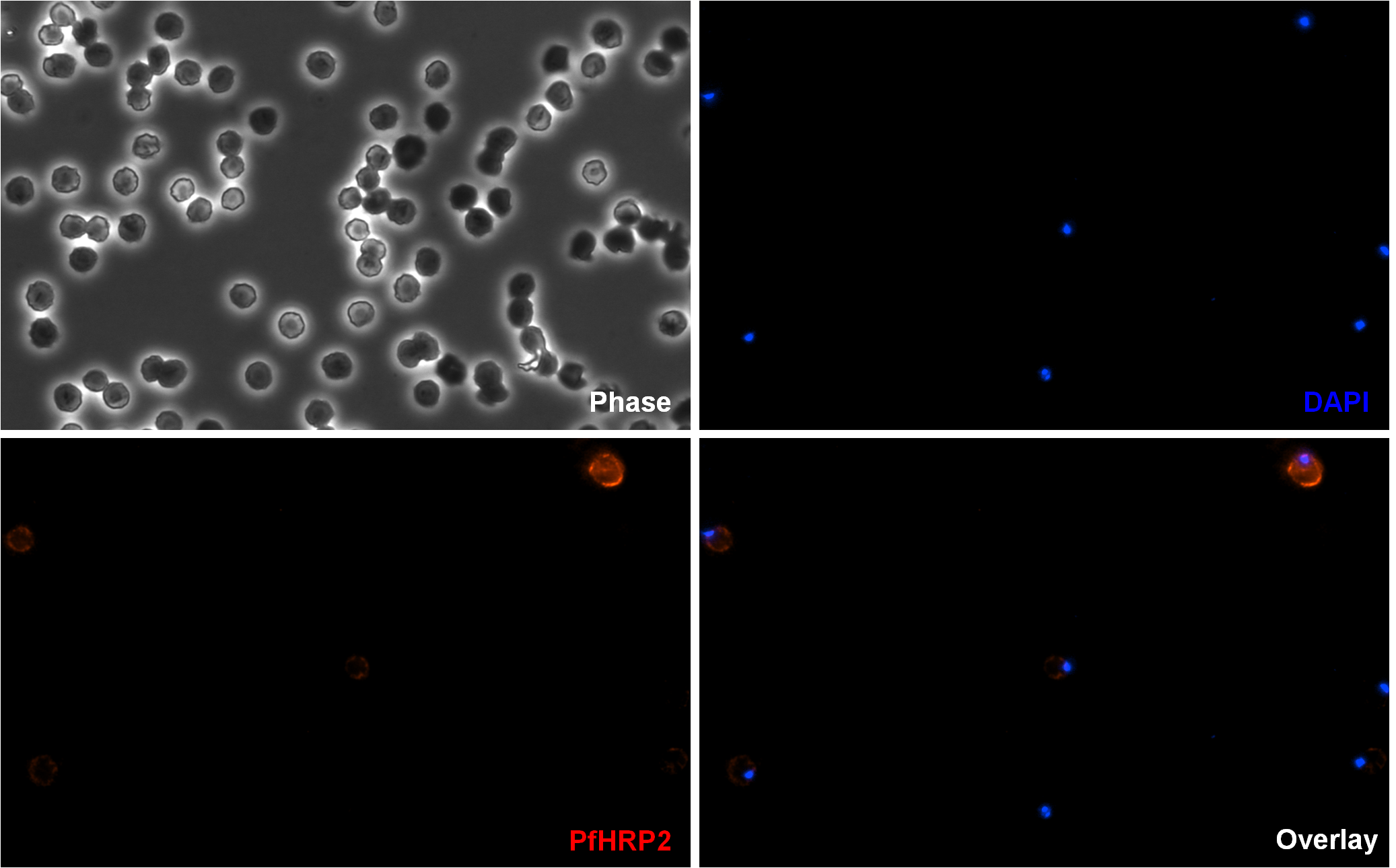

Supplement: Supplementary file 4 — Additional file 4: Figure S3. Investigation of the presence of once-infected RBCs pre-treatment. iRBCs were isolated from a P. falciparum infected patient with a parasitaemia of 2.9% quantified by blood film prior to receiving treatment. No once-infected RBCs were observed prior to treatment. iRBCs were defined as cells with positive PfHRP2 (red) and DAPI staining (blue). Once-infected RBCs were defined as cells with positive PfHRP2 staining (red) but no DAPI staining (blue). [file 12936_2019_2712_MOESM4_ESM.tif]

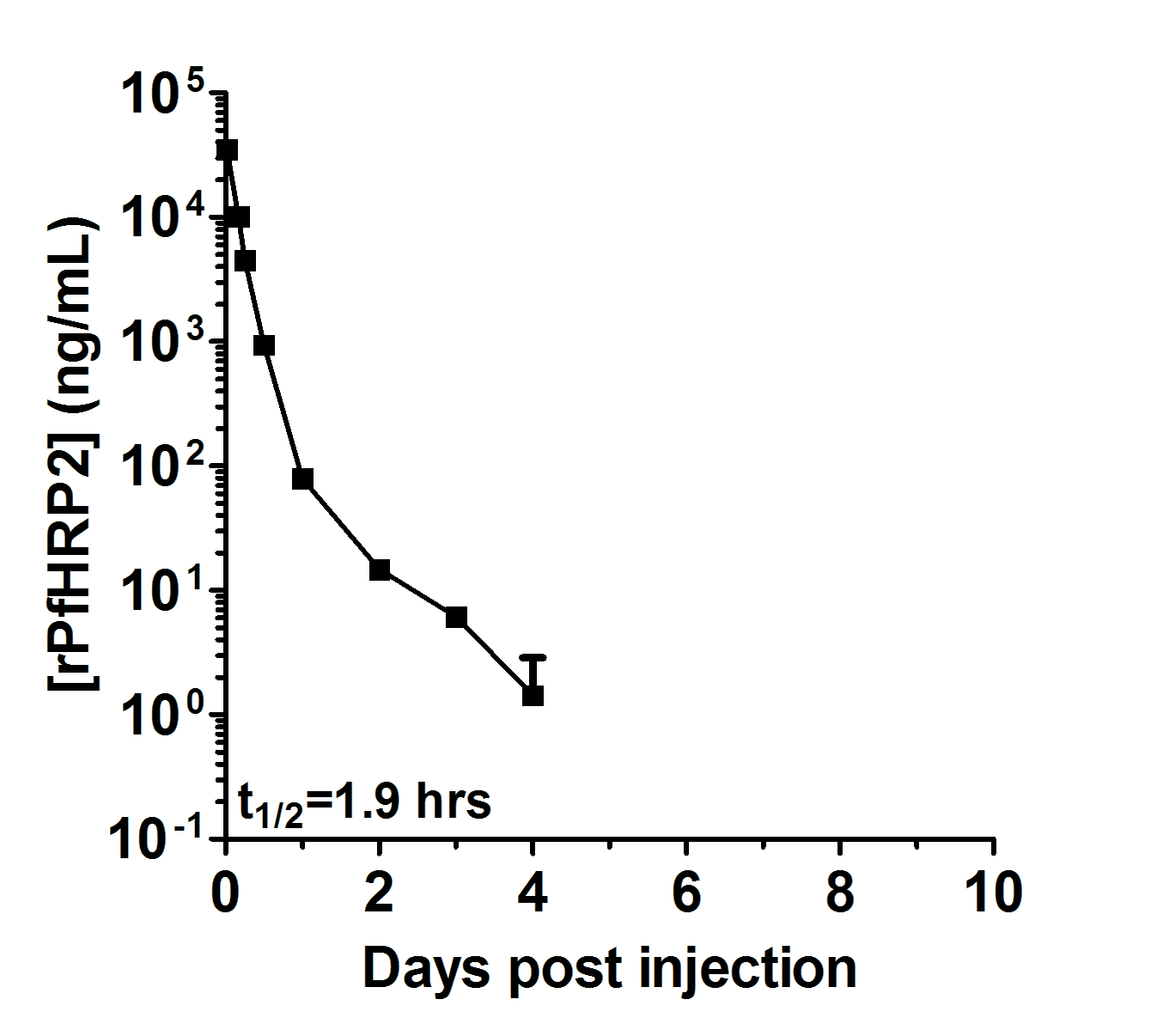

Supplement: Supplementary file 7 — Additional file 7: Figure S4. Kinetics of recombinant PfHRP2 in plasma following protein injection. Female BALB/c mice (n = 3) were injected with rPfHRP2 protein. Plasma was collected at regular intervals post injection for quantification of rPfHRP2 plasma concentration. The protein half-life in each mouse was calculated and averaged using a first-order decay equation model constrained at a plateau of Y = 0. Data are represented as mean ± SEM. [file 12936_2019_2712_MOESM7_ESM.tif]
