# Supplementary material for: In vivo compartmental kinetics of Plasmodium falciparum histidine-rich protein II in the blood of humans and in BALB/c mice infected with a transgenic Plasmodium berghei parasite expressing histidine-rich protein II
Source: Malar J. 2019 Mar 13;18:78. doi: 10.1186/s12936-019-2712-3 (PMC6416945; doi:10.1186/s12936-019-2712-3)
Supplement: Supplementary file 5 — Additional file 5: Table S2. Distribution of PfHRP2 between naïve human or mouse RBCs and PfHRP2-containing plasma/sera incubated in vitro. [file 12936_2019_2712_MOESM5_ESM.docx]

**Additional** **Table S2.** Distribution of PfHRP2 between naïve human or mouse RBCs and PfHRP2-containing plasma/sera incubated *in vitro*.

| **Source of plasma/ serum** | **[RBC] (ng/mL)** | **[Plasma/Serum] (ng/mL)** | **[RBCs]: [plasma/serum]** | **Bound (%)** |
| --- | --- | --- | --- | --- |
| Plasma isolated from *P. falciparum* infected human patients (n=5) | 2 ± 1 | 1,827 ± 294 | 0.0011 | 0.11 |
| Human serum + rPfHRP2 (n=3) | 679 ± 517 | 154,086 ± 54,961 | 0.0034 | 0.34 |
|  | 6 ± 0.2 | 27,246 ± 11,041 | 0.00031 | 0.030 |
| Mouse plasma + rPfHRP2 (n=3) | 631 ± 195 | 176,601 ± 24,676 | 0.0035 | 0.35 |
|  | 49 ± 20 | 11,099 ± 1,642 | 0.0048 | 0.48 |

Data are represented as means or mean ± SEM.
