# Supplementary material for: In vivo compartmental kinetics of Plasmodium falciparum histidine-rich protein II in the blood of humans and in BALB/c mice infected with a transgenic Plasmodium berghei parasite expressing histidine-rich protein II
Source: Malar J. 2019 Mar 13;18:78. doi: 10.1186/s12936-019-2712-3 (PMC6416945; doi:10.1186/s12936-019-2712-3)
Supplement: Supplementary file 6 — Additional file 6: Table S3. Distribution of rPfHRP2 between RBC and plasma fractions isolated from mice (n = 3) post-recombinant protein injection. [file 12936_2019_2712_MOESM6_ESM.docx]

**Additional Table S3.** Distribution of rPfHRP2 between RBC and plasma fractions isolated from mice (n=3) post-recombinant protein injection.

| **Hours post rPfHRP2 injection** | **[RBC] (ng/mL)** | **[Plasma] (ng/mL)** | **[RBC]:[Plasma]** | **Bound (%)** |
| --- | --- | --- | --- | --- |
| 0.5 | 13 ± 6 | 35,113 ± 2974 | 0.00036 | 0.036 |
| 6 | 3 ± 1 | 4,501 ± 403 | 0.00063 | 0.063 |
| 12 | 12 ± 6 | 941 ± 157 | 0.011 | 1.09 |
| 24 | 0 | 79 ± 4 | 0 | 0 |

Data are represented as means or mean ± SEM.
